
JOURNAL INFORMATION
==============================
NLM Title Abbreviation: JBRA Assist Reprod
Journal ID: JBRA Assist Reprod
NLM Journal ID: 101684552
Journal ID: jbra

JBRA Assisted Reproduction

ISSN: 1517-5693
EISSN: 1518-0557
Publisher: Brazilian Society of Assisted Reproduction

ARTICLE INFORMATION
==============================
PMCID: PMC6106626
PMID: 30129353
DOI: 10.5935/1518-0557.20180063
Article version: 1
Subjects: Oral Presentations

Abstracts of the 22st Annual Congress of the SBRA, Brasilia/DF, 01-04 August 2018

Publication date: 2018 Jul-Sep
Print publication date: 2018 Jul-Sep
Volume: 22
Issue: 3
First page: 263
Last page: 266
License: This is an Open Access article distributed under the terms of the Creative Commons Attribution License, which permits unrestricted use, distribution, and reproduction in any medium, provided the original work is properly cited.
License URL: https://creativecommons.org/licenses/by/4.0/

==============================


O-01. Ovarian response to stimulation and suboptimal endometrial development are associated with adverse perinatal outcomes in intracytoplasmic sperm injection cycles

Borges Jr. E. 1 2
Zanetti B.F. 1 2
Braga D.P.A.F. 1 2
Setti A.S. 1 2
Figueira R.C.S. 1
Iaconelli Jr. A. 1 2
1 Fertility Medical Group, São Paulo, SP - Brazil.
2 Instituto Sapientiae - Centro de Estudos e Pesquisa em Reprodução Assistida, SP - Brazil.

O-02. Should single embryo transfer to be used for patients with any kind of infertility factor? Preliminary outcomes

Monteleone P.A.A. 1 2
Petersen P.G.M.F. 1
Peregrino P.F.M. 1 2
Miorin J. 1
Gomes A.P. 1
Fujii M.G. 1
Martin H. de 1 2
Bonetti T.C.S. 3
Gonçalves S.P. 1 2
1 Centro de Reprodução Humana Monteleone, Brazil.
2 Disciplina de Ginecologia - Departamento de Obstetrícia e Ginecologia. Faculdade de Medicina da Universidade de São Paulo, Brazil.
3 Departamento de Ginecologia. Escola Paulista de Medicina da Universidade Federal de São Paulo, Brazil.

O-03. Serum metabolites as molecular predictive markers of ovarian response to controlled stimulation: a pilot study

Borges Jr. E. 1 2
Montani D.A. 3
Setti A.S. 1 2
Zanetti B.F. 1 2
Figueira R.C.S. 1
Iaconelli Jr. A. 1 2
Silva D.O. 3
Braga D.P.A.F. 1 2
1 Fertility Medical Group, São Paulo, SP - Brazil.
2 Instituto Sapientiae - Centro de Estudos e Pesquisa em Reprodução Assistida, SP - Brazil.
3 Grupo de Bio-orgânica e Bioanalítica - Instituto de Ciências Ambientais, Químicas e Farmacêuticas-UNIFESP, Brazil.

O-04. The effects of overweight and obesity on Assisted Reproduction Technology outcomes

Silvestrim R.L. 1
Bos-Mikich A. 1
Kulmann M.I.R. 1
Frantz N. 1
1 Nilo Frantz Reproductive Medicine, Porto Alegre, Brazil.

O-05. Evaluation of the influence of immunological factors and trombofilia factors in the decrease of the ovarian reserve

Bilibio J.P. 1 2 3
Lopes B. 1 3
Nascimento I. 1 3
Meireles A. 2
Gama T.B. 1 3
Lorenzzoni P.L. 2 3
1 Universidade Federal do Pará, BELEM - PA - Brazil.
2 Clinica PRONATUS - Medicina Reprodutiva, BELÉM - PA - Brazil.
3 Grupo de Pesquisa BILIBIO.

O-06. Prevalence of Human Papillomavirus (HPV) in semen of patients submitted to assisted reproduction in a private clinic in Brazil

Bossi R. L. 1
Valadares J.B.F. 2
Del Puerto H.L. 3
Rocha M.G.L. 4
Braga L.C. 5
Sampaio M.A.C. 1
França P.P. 1
Alvarenga D.M. 1
Geber S. 1
1 Origen - Centro de Medicina Reprodutiva- Belo Horizonte/MG - Brazil.
2 Centro Universitário UNA- Belo Horizonte/MG - Brazil.
3 Departamento de Patologia Geral do Instituto de Ciências Biológicas da Universidade Federal de Minas Gerais - Brazil.
4 Departamento de Análises Clinicas e Toxicológicas da Faculdade de Farmácia da Universidade Federal de Minas Gerais - Brazil.
5 FUNED - Fundação Ezequiel Dias- Belo Horizonte/MG - Brazil.

O-07. Investigation of causes of recurrent abortion in women submitted to in vitro fertilization after natural pregnancy

Bilibio J.P. 1 2 3
Nascimento I. 1
Gama T.B. 1 3
Lopes B. 1 3
Meireles A. 2
Lorenzzoni P.L. 2 3
1 Universidade Federal do Pará, BELEM - PA - Brazil.
2 Clinica PRONATUS - Medicina Reprodutiva, BELÉM - PA - Brazil.
3 Grupo de Pesquisa BILIBIO.

O-08. Embryo placement in IVF and reproductive outcomes: a cohort analysis and review

Santos M.M. dos 1
Silva A.A. 1
Barbosa A.C.P. 1
Brum G. 1
Nakagawa H.M. 1
Cabral I. 1
Iglesias J.R. 1
Barbosa M.W.P. 1
1 Genesis - Centro de Assistência em Reprodução Humana.

